# Oral contraceptive use and anterior cruciate ligament injury or reconstruction in women: A systematic review and meta‐analysis of very low‐certainty evidence

**DOI:** 10.1002/jeo2.70940

**Published:** 2026-10-06

**Authors:** Eiki Nicholas Kobayashi, Natalia Zampa Silva, Laura Amaral Coelho de Azevedo, Maria Eugênia Cammarota de Abreu, Juliano Mangini Dias Malpaga, Diego Escudeiro de Oliveira, Pedro Baches Jorge

**Affiliations:** ^1^ Faculdade de Ciências Médicas da Santa Casa de São Paulo, São Paulo São Paulo Brazil; ^2^ Department of Orthopedics and Traumatology Irmandade da Santa Casa de Misericórdia de São Paulo, São Paulo São Paulo Brazil; ^3^ Faculdade de Ciências Médicas e da Saúde Pontifícia Universidade Católica de São Paulo, Sorocaba São Paulo Brazil

**Keywords:** anterior cruciate ligament injuries, anterior cruciate ligament reconstruction, meta‐analysis, oral contraceptives, risk factors, women

## Abstract

**Purpose:**

To determine whether oral contraceptive use is associated with anterior cruciate ligament injury or reconstruction in women and to examine whether associations vary according to age, contraceptive formulation, duration of use and outcome definition.

**Methods:**

PubMed, Scopus, Embase and Latin American and Caribbean Health Sciences Literature (LILACS) were searched through 31 July 2026 (LILACS rerun with multilingual terms on 16 September 2026). Comparative observational studies were meta‐analysed. The primary analysis pooled adjusted or matched odds ratios (ORs) for reconstruction or operatively treated injury using random‐effects restricted maximum‐likelihood models with Hartung–Knapp–Sidik–Jonkman confidence intervals (CIs). Risk of bias was assessed with risk of bias in nonrandomised studies of exposures and certainty with grading of recommendations assessment, development and evaluation.

**Results:**

Ten studies were included; seven were meta‐analysed. The primary analysis (four studies) showed no clear association (OR, 0.89; 95% CI, 0.77–1.02; *I* ^2^ = 84%; *τ* ^2^ = 0.01; 95% prediction interval, 0.66–1.19). Two crude athlete studies and the active‐comparator cohort (hazard ratio, 0.95; 95% CI, 0.89–1.01) also had CIs including 1. Adding one crude database estimate yielded OR 0.85 (95% CI, 0.74–0.98; *I* ^2^ = 90%; 95% prediction interval, 0.61–1.19). Leave‐one‐out results were unstable. The certainty of evidence was very low.

**Conclusions:**

The primary adjusted or matched analysis did not establish a clear association between oral contraceptive use and anterior cruciate ligament reconstruction or operatively treated injury; substantial heterogeneity, residual confounding and very low certainty of evidence preclude causal interpretation. Current evidence does not support initiating or selecting oral contraceptives for anterior cruciate ligament injury prevention. Well‐designed prospective studies with standardised exposure and outcome definitions are needed.

**Level of Evidence:**

Level IV, systematic review of observational studies.

**Abbreviations**
ACL: anterior cruciate ligament
BMI: body mass index
CI: confidence interval
CPT: current procedural terminology
EE: ethinyl estradiol
GRADE: grading of recommendations assessment, development and evaluation
HC: hormonal contraceptive
HKSJ: Hartung‐Knapp‐Sidik‐Jonkman
HR: hazard ratio
ICD‐9‐CM: International Classification of Diseases, Ninth Revision, Clinical Modification
IUD: intrauterine device
IV: inverse variance
LILACS: Latin American and Caribbean Health Sciences Literature
NCAA: National Collegiate Athletic Association
OC: oral contraceptive
OCP: oral contraceptive pill
OR: odds ratio
PICO: population, intervention/exposure, comparator and outcome
PRISMA: preferred reporting items for systematic reviews and meta‐analyses
PROSPERO: International Prospective Register of Systematic Reviews
REML: restricted maximum likelihood
ROBINS‐E: risk of bias in nonrandomised studies of exposures
RR: relative risk
SE: standard error

## INTRODUCTION

Anterior cruciate ligament (ACL) rupture is one of the most common and clinically relevant ligamentous injuries of the knee, particularly among young and physically active individuals [19]. ACL injury often results in prolonged absence from sports, frequently requires surgical treatment and is associated with an increased risk of long‐term sequelae, including early knee osteoarthritis [8]. Given its functional, athletic and socioeconomic impact, identifying modifiable risk factors for ACL injury remains an important focus of sports medicine research.

Female athletes have a substantially higher risk of ACL injury than male athletes participating in comparable sports. Epidemiological studies have reported that women may sustain ACL injuries at rates approximately three to six times higher than men, particularly in sports involving pivoting, cutting, jumping and rapid deceleration [2, 19]. This sex‐based disparity is multifactorial and has been attributed to anatomical differences, neuromuscular control patterns, movement biomechanics and hormonal influences [12, 21].

Among the hormonal factors potentially involved in ACL injury susceptibility, cyclic fluctuations in estrogen and progesterone across the menstrual cycle have received considerable attention. Experimental studies suggest that estrogen may affect collagen metabolism and the mechanical properties of the ACL, while clinical and observational investigations have reported variations in knee laxity and ACL injury occurrence across different phases of the menstrual cycle [16, 27, 28]. These findings provide a biologically plausible rationale for investigating whether hormonal modulation may influence ACL injury risk in women.

Oral contraceptives (OCs) have been proposed as a potential hormonal‐modulating factor that may influence ACL injury risk. By suppressing ovulation and reducing endogenous hormonal fluctuations, combined OCs may attenuate cyclical changes in ligamentous laxity and knee stability. This mechanism has led to the hypothesis that OC use could be associated with a lower risk of ACL injury or ACL reconstruction in women of reproductive age [20, 22].

Several observational studies and population‐based analyses have evaluated the association between OC use and ACL injury or reconstruction, but their findings remain inconsistent. Some studies have reported lower odds or risk of ACL injury or reconstruction among OC users, whereas others have found no clear association or have reported results that vary according to age, exposure definition, contraceptive formulation or outcome ascertainment [5, 6, 7, 11, 20, 22]. Differences in study design, population characteristics, comparator groups, adjustment for confounders and whether the outcome was defined as ACL injury or ACL reconstruction may partly explain the heterogeneity across studies.

Previous reviews have explored the relationship between menstrual‐cycle phase, hormonal contraceptive use, ACL laxity and ACL injury risk [10]. Paska et al. reported a pooled estimate of 0.88 (95% confidence interval [CI], 0.77–1.01), with very high heterogeneity [18]. The present review refines this evidence through an updated OC‐specific search, separation of injury and reconstruction outcomes, prioritisation of adjusted or matched estimates and assessment of heterogeneity and certainty; crossing a statistical‐significance threshold does not establish a new clinical finding.

The purpose of this study was to determine whether OC use is associated with anterior cruciate ligament injury or reconstruction in women and to examine whether associations vary according to age, contraceptive formulation, duration of use and outcome definition. We hypothesised that OC use would be associated with lower odds of ACL injury or reconstruction; this hypothesis does not imply a causal preventive effect.

## METHODS

### Protocol and registration

This systematic review and meta‐analysis were conducted and reported in accordance with the preferred reporting items for systematic reviews and meta‐analyses (PRISMA) guidelines and registered on the International Prospective Register for Systematic Reviews (PROSPERO; CRD420251165678).

### Study identification and selection

MEDLINE via PubMed, Scopus and Embase were searched on 31 July 2026. LILACS was rerun on 16 September 2026 using an expanded DeCS‐aligned strategy with Spanish, Portuguese and English terms. No restrictions were applied regarding publication date or language. The complete search strategies for each database are provided in Table S1. Retrieved records were imported into Zotero for duplicate removal and subsequently screened using Rayyan.

Study selection was performed independently by two reviewers. Titles and abstracts were screened first, followed by full‐text assessment of potentially eligible studies. Disagreements were resolved by discussion and consensus; when consensus could not be reached, a third reviewer was consulted. The study selection process was documented using a PRISMA flow diagram.

The review question was structured according to the population, intervention/exposure, comparator and outcome framework. The population of interest consisted of women of reproductive age, approximately 13 to 49 years old, who were physically active or included in population‐based datasets with available ACL injury or reconstruction outcomes. The exposure was OC use, regardless of formulation, dose, duration or timing of exposure. The comparator was nonuse of OCs or no hormonal contraceptive exposure, according to the definition used in each included study. Outcomes were distinguished as clinically identified ACL injury, ACL reconstruction, or operatively treated ACL injury, as defined by clinical diagnosis, imaging, surgical records, registry data or administrative coding. The primary quantitative analysis focused on reconstruction or operatively treated injury; these outcomes were not assumed to be interchangeable with injury incidence.

Eligible studies included observational studies and clinical trials evaluating OC exposure and ACL injury or reconstruction outcomes. Comparative studies were required to include OC users and an appropriate nonuser or nonhormonal‐contraceptive group. Case reports, narrative reviews, systematic reviews, editorials, letters, conference abstracts and studies without relevant ACL outcomes were excluded. Studies limited to patients with ACL injury or reconstruction, without a non‐ACL control group or data permitting estimation of the exposure–outcome association, were retained only for qualitative synthesis when they informed prespecified secondary questions and were excluded from quantitative meta‐analysis.

### Data extraction

One reviewer extracted data from the included studies using a standardised spreadsheet. Extracted data included first author, year of publication, country, study design, data source or setting, study period, population characteristics, sample size, age range or mean age, OC exposure definition, comparator definition, outcome definition, type of ACL‐related outcome, reported effect estimates, 95% CIs, adjustment status, variables included in adjusted models when available, main findings and limitations relevant to this review. For studies not eligible for quantitative synthesis, the reasons for exclusion from meta‐analysis were recorded. Authors of the included studies were contacted to request missing or unclear data when necessary.

### Risk of bias assessment

Two independent reviewers assessed the risk of bias of the included studies, with disagreements resolved by consensus or consultation with a third reviewer. Risk of bias was assessed using the Risk of bias in nonrandomised studies of exposures (ROBINS‐E) tool [13], as all included studies evaluated nonrandomized exposure‐outcome associations. Studies without an uninjured or non‐ACL comparison group were judged to be at high risk of bias due to confounding because they could not address the association of interest.

ROBINS‐E evaluates bias across seven domains: confounding, measurement of exposure, selection of participants, postexposure interventions, missing data, measurement of outcomes and selection of the reported result. Each domain was judged according to ROBINS‐E guidance, and an overall risk‐of‐bias judgement was assigned for each study as low risk, some concerns, high risk, very high risk or no information. Assessments focused on the association between OC use and ACL injury or reconstruction, with particular attention to confounding factors such as age, sport or activity level, prior knee injury, body mass index and other factors potentially associated with both contraceptive use and ACL injury risk.

### Certainty of evidence assessment

The certainty of evidence for the primary outcome was assessed using the grading of recommendations assessment, development and evaluation (GRADE) approach [9]. The assessment considered risk of bias, inconsistency, indirectness, imprecision and potential publication bias across the body of evidence. Because all studies included in the quantitative synthesis were observational, the initial certainty of evidence was considered low. Certainty could be downgraded for serious or very serious concerns in any GRADE domain or upgraded when appropriate, according to GRADE guidance. The overall certainty of evidence was classified as high, moderate, low or very low.

Two independent reviewers conducted the GRADE assessment. Disagreements were resolved by discussion and consensus; when necessary, a third reviewer was consulted.

### Data analysis

The primary quantitative outcome was the association between OC use and ACL reconstruction or operatively treated ACL injury. Odds ratios (ORs) were used as the common effect measure, and adjusted estimates were preferred. When a study reported a risk ratio and the outcome was rare, the estimate was treated as an approximation of the OR for pooling; hazard ratios (HRs) were not pooled. When adjusted estimates were unavailable but sufficient 2 × 2 data were provided, crude ORs were calculated from the numbers of events and nonevents in the exposed and nonexposed groups.

For quantitative synthesis, effect estimates were transformed to the natural logarithm of the OR [log(OR)] and analysed using the generic inverse variance method. Standard errors were derived from reported 95% CIs when necessary. Random‐effects meta‐analyses were performed using restricted maximum‐likelihood (REML) estimation for between‐study variance, and CIs were calculated using the Hartung‐Knapp‐Sidik‐Jonkman (HKSJ) method. Results were back‐transformed and presented as ORs with 95% CIs.

The primary meta‐analysis was restricted to the four studies providing adjusted or matched estimates for reconstruction or operatively treated ACL injury. Crude estimates were analysed separately. Clinically identified ACL injury estimates and the active‐comparator cohort HR were presented individually without pooling incompatible designs or effect measures. Statistical heterogeneity was evaluated using Cochran's *Q*, *I* ^2^, *τ* ^2^ and 95% prediction intervals; prediction intervals were calculated with a *t* distribution with *k* − 1 degrees of freedom, as implemented in Review Manager Web. Because clinical and methodological heterogeneity was expected, random‐effects models were used for all pooled analyses.

A sensitivity analysis added the crude Fry estimate to the four adjusted or matched reconstruction estimates. A further sensitivity analysis retained the original seven‐study synthesis combining crude and adjusted estimates and different outcome definitions; it was not used as the primary result. Leave‐one‐out analyses evaluated influence using the same log estimates and standard errors as the RevMan analyses (Table S5).

Age‐stratified findings were summarised narratively. Study‐specific estimates for women aged 15–19 years were displayed in an effect‐estimate plot without calculating a pooled estimate because of differences in exposure definitions, analytical adjustment, outcome ascertainment and potential population overlap across administrative database studies. Studies reporting age‐specific incidences without age‐specific effect estimates or sufficient denominators for exact calculation were described narratively.

Findings related to OC formulation, duration of use and timing of exposure were also synthesised narratively because of heterogeneous exposure definitions and insufficient comparable data for pooling. Additional secondary outcomes planned in the protocol, including ACL reinjury or contralateral ACL injury, knee laxity, knee function, other knee injuries, hormonal levels and markers of collagen synthesis or metabolism, were synthesised narratively when available.

Meta‐analyses were conducted using Review Manager Web. A two‐sided *p* < 0.05 was considered statistically significant. Leave‐one‐out models were independently calculated from the RevMan input values. Publication‐bias testing was not performed because fewer than 10 studies contributed to each synthesis.

## RESULTS

### Study selection

The database search identified 249 records, including 80 from PubMed, 10 from Scopus and 159 from Embase. The expanded DeCS‐aligned LILACS rerun identified no records. This zero yield was considered a possible indexing or search‐sensitivity limitation and was not interpreted as evidence that no relevant Latin American literature exists. After removal of 68 duplicates, 181 records underwent title and abstract screening, of which 155 were excluded. Twenty‐six reports were assessed in full text.

Of these, 16 were excluded because they were not primary studies (*n* = 3), did not assess ACL injury incidence (*n* = 6), did not compare ACL injury outcomes between hormonal contraceptive users and nonusers (*n* = 3) or were not completed studies (*n* = 4). Thus, 10 studies [1, 3, 5, 6, 7, 11, 15, 20, 22, 24] were included in the qualitative synthesis, of which 7 provided sufficient comparative data for quantitative meta‐analysis. The study selection process is presented in Figure 1.

**Figure 1:**
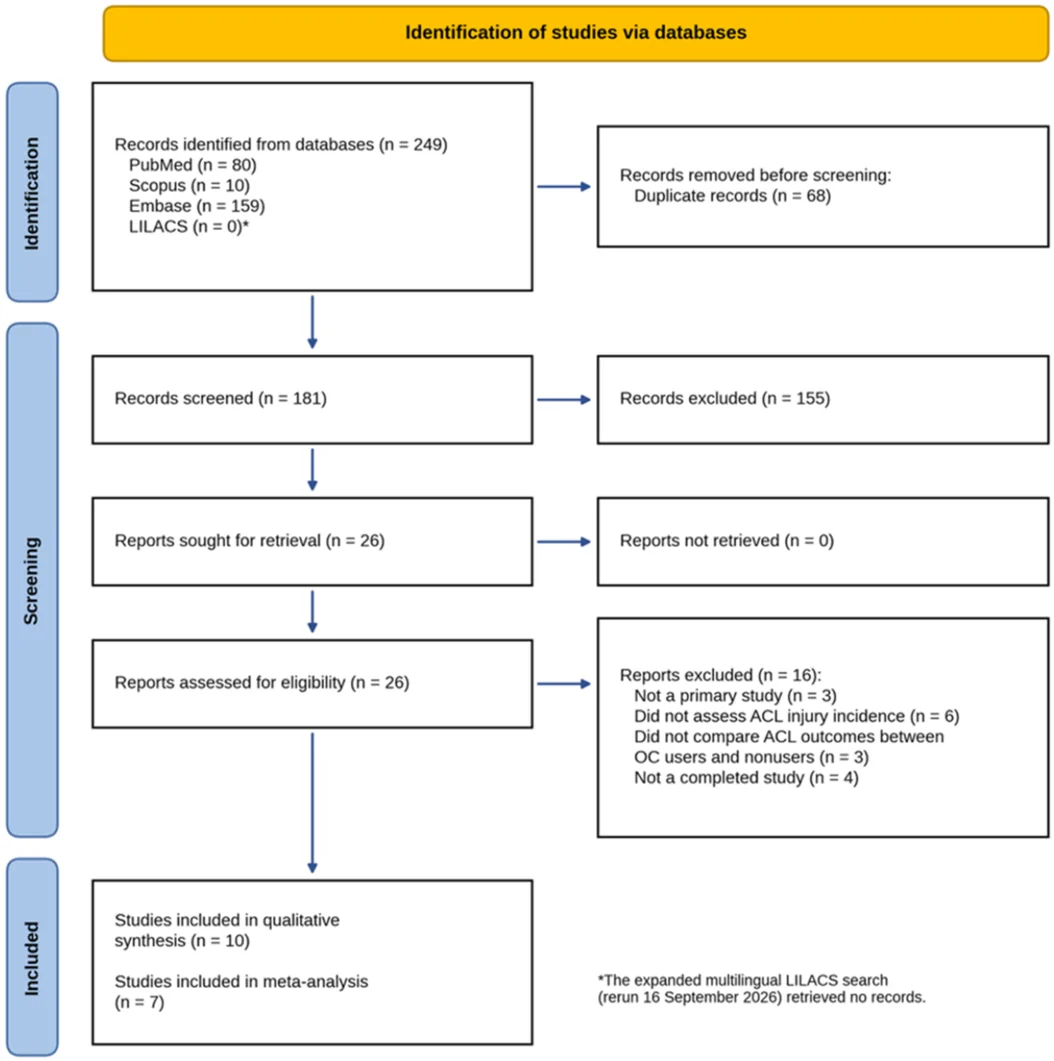
PRISMA flow diagram of study identification and selection. Figure 1. PRISMA 2020 flow diagram showing the identification, screening, eligibility assessment and inclusion of studies evaluating the association between oral contraceptive use and anterior cruciate ligament injury or reconstruction. ACL, anterior cruciate ligament; LILCAS, Latin American and Caribbean Health Sciences Literature; PRISMA, preferred reporting items for systematic reviews and meta‐analyses.

### Study characteristics

The characteristics of the included studies are summarised in Table 1. Ten studies published between 2002 and 2025 were included. Study designs comprised case‐control studies, retrospective comparative database studies, prospective observational studies and injury‐surveillance studies.

**Table 1:** Characteristics of the included studies evaluating oral contraceptive use and anterior cruciate ligament injury or reconstruction.

Author/year	Country	Design	Population	Sample size	Exposure	Comparator	Outcome	Effect estimate	Main findings
Rahr‐Wagner et al., 2014 [20]	Denmark	Nationwide population‐based case‐control study	Women with incident operatively treated ACL injury/reconstruction and age‐matched population controls	4497 cases; 8858 controls	Low‐dose oral contraceptive use based on redeemed prescriptions during the 5‐year period before the index date; main comparison: ever users (≥ 1 redeemed prescription)	Never users: no redeemed OC prescriptions during the 5‐year period before the index date	Operatively treated ACL injury identified through primary ACL reconstruction in the Danish Knee Ligament Reconstruction Registry	Adjusted RR 0.82 (ever users vs. never users) (0.75–0.90)	Ever OC use was associated with a lower likelihood of operatively treated ACL injury compared with never use.
Gray et al., 2016 [7]	United States	Matched case‐control study using insurance claims	Commercially insured women aged 15–39 years with surgical reconstruction of the cruciate ligaments and matched controls	12,819 cases; 38,457 controls (3:1 matching)	Any oral contraceptive prescription fill in the 12 months before index date	No oral contraceptive prescription fill in the 12 months before index date	Surgical reconstruction of the cruciate ligaments identified by ICD‐9‐CM procedure codes 81.43 (triad knee repair) and 81.45 (other repair of the cruciate ligaments); authors cite that 99.3% of outpatient 81.45 codes correspond to ACL reconstruction	Adjusted OR 0.987 (any OC use vs. nonuse, all ages) (0.94–1.04)	No significant overall association between any OC use and ACL reconstruction; age‐stratified analyses showed lower odds in ages 15–19 and higher odds in some older age groups (25–34 years).
DeFroda et al., 2019 [5]	United States	Retrospective matched database study	Female patients aged 15–49 years in matched OC and non‐OC cohorts	82,874 OC users; 82,874 non‐OC users; ACL reconstructions: 465 in OC group and 569 in non‐OC group	Initiation of common combination (estrogen + progesterone) oral contraceptive pills within 1 year before ACL injury and still active at the time of injury, identified through prescription records	Matched female patients not taking OCs (matched on age, BMI and Charlson Comorbidity Index)	ACL tear requiring ACL reconstruction, identified by CPT code 29888	OR: 0.82 (OC use vs non‐OC use, all ages) (0.72–0.92); RevMan reconstructed CI: 0.73–0.93	OC users had lower odds/rate of ACL reconstruction than matched non‐OC users; the inverse association was driven primarily by the 15–19‐year age group (OR 0.37; 0.27–0.50).
Herzog et al., 2020 [11]	United States	Active comparator new‐user cohort study with case‐control replication analysis	Women aged 13–45 years in claims data; primary cohort compared new low‐dose OC initiators with new IUD users; secondary analysis replicated case‐control ever/never OC designs (1‐ and 5‐year look‐backs)	Cohort: 2,370,286 OC initiators and 621,798 IUD users; 5‐year case‐control replication: 11,022 cases and 22,044 age‐matched controls	For the meta‐analysis: at least one claim for low‐dose OC during the 5 years before ACL reconstruction in the case‐control replication	No OC claims during the same 5‐year look‐back window	Incident ACL reconstruction identified by CPT code 29888 in the case‐control replication	OR: 0.90 (ever vs.never low‐dose OC use, 5‐year case‐control replication) (0.85–0.94); RevMan reconstructed CI: 0.86–0.95	The active comparator cohort suggested no association (adjusted HR: 0.95; 95% CI: 0.89–1.01), whereas the 5‐year case‐control replication showed a modest inverse association (OR: 0.90; 95% CI: 0.85–0.94) and the 1‐year replication showed OR 0.96 (0.94–0.99).
Agel et al., 2006 [1]	United States	Prospective observational cohort/injury surveillance study	Female collegiate basketball and soccer athletes from participating NCAA schools	3150 athletes; 1024 reported hormonal therapy and 2026 reported no hormonal therapy; 45 noncontact ACL injuries (16 in users vs. 29 in nonusers)	Hormonal therapy/oral contraceptive use recorded on team rosters at the start and end of season; OCs classified as monophasic or triphasic when applicable	No hormonal therapy	Noncontact ACL injury sustained during the NCAA season, identified by certified athletic trainer or team physician	Crude OR: 1.09 calculated from 16/1024 vesus 29/2026 noncontact ACL injuries (0.59–2.04)	No difference in noncontact ACL injury rate between athletes using hormonal therapy and those not using hormonal therapy.
Ruedl et al., 2009 [22]	Austria	Case‐control study	Female recreational alpine skiers with noncontact ACL injury and age‐matched uninjured female skier controls	93 cases; 93 controls	Self‐reported current oral contraceptive use (formulation type not specified)	No current oral contraceptive use	Noncontact ACL injury sustained during recreational alpine skiing; MRI was used for diagnosis	Crude OR: 0.95 calculated from 32/93 cases and 33/93 controls using OC (0.52–1.74)	OC use rates were similar between ACL‐injured skiers (34.4%) and controls (35.5%); no significant association was detected.
Fry et al., 2025 [6]	United States	Retrospective comparative database study using Colorado Health Data Compass	Female patients aged 15–35 years with ACL injury requiring reconstruction compared with female patients without a history of ACL injury or reconstruction	OC cohort: 745,062 women, 654 ACL reconstructions; no‐contraceptive cohort: 12,766,138 women, 15,253 ACL reconstructions	Total OC use (norethindrone‐only, drospirenone + EE, norethindrone + EE and norgestimate + EE) based on selected RxNorm medication codes; contraceptive use documented up to 6 months before ACL reconstruction	No contraceptive use	ACL injury requiring arthroscopic ACL reconstruction, identified by CPT code 29888	Crude OR 0.73 calculated from 654/745,062 versus 15,253/12,766,138 (0.68–0.79)	OC use was associated with lower crude incidence/odds of ACL reconstruction compared with no contraceptive use; the strongest association was observed for progestin‐only OCs (norethindrone only).
Wang et al., 2023 [24]	United States	Retrospective comparative risk‐factor analysis within an ACL reconstruction cohort	Patients undergoing ACL reconstruction; OC use was compared within the female ACL reconstruction subgroup, without a non‐ACL comparison group	11,498 ACL reconstructions total; 5967 in females; 1416 (23.7%) OC users among female ACL reconstruction patients	OC use prior to and at the time of ACL reconstruction, identified by USC codes 33210 and 33230	Female ACL reconstruction patients not taking OCs; no women without ACL injury or reconstruction	ACL reconstruction identified by CPT code 29888	No effect estimate for incident ACL injury or reconstruction; within‐cohort comparison only	Within the ACL reconstruction cohort, OC users were younger than nonusers (mean, 26.17 vs. 33.58 years; *p* < 0.001). Because all participants underwent ACL reconstruction, the study did not estimate incident ACL injury or reconstruction risk versus a non‐ACL comparison group.
Lefevre et al., 2013 [15]	France	Prospective observational case series	Women with ACL tear sustained during recreational skiing; postmenopausal patients and those with irregular cycle/LMP > 30 days were excluded	172 women with ACL tear included in analysis	Contraceptive method, including oral contraceptive use, self‐reported in a questionnaire	No non‐ACL control group; only within‐case contraceptive comparisons	ACL tear sustained during recreational skiing, diagnosed clinically by mountain physicians	Not applicable for OC vs non‐OC ACL risk; no risk/odds estimate provided	53/172 (30.8%) women used oral contraceptives; ACL tears were more frequent in the preovulatory phase than in the postovulatory phase; OC use was similar across menstrual phases.
Arendt et al., 2002 [3]	United States	Descriptive observational study of injured athletes	Female collegiate athletes who sustained noncontact ACL injury and had menstrual/OC information available	128 injured athletes identified; 83 eligible for analysis (25 ‘On‐Pills’ and 58 ‘Off‐Pills’)	Oral contraceptive use status among injured athletes	No oral contraceptive use among injured athletes; no uninjured denominator available	Noncontact ACL injury in female collegiate athletes	Not applicable for OC vs non‐OC ACL risk; no denominator for uninjured athletes	The study assessed periodicity of noncontact ACL injuries and described differences by OC status among injured athletes; both groups showed periodicity around the menstrual cycle.

*Note*: Table 1 Characteristics of the 10 studies included in the qualitative synthesis. Seven studies provided directly reported effect estimates or sufficient raw data to calculate crude odds ratios and were included in the quantitative synthesis, whereas three studies were retained for qualitative synthesis only because they did not provide an appropriate comparison group or sufficient comparative data to estimate the association between oral contraceptive use and ACL injury or reconstruction.

Abbreviations: ACL, anterior cruciate ligament; BMI, body mass index; CI, confidence interval; CPT, current procedural terminology; EE, ethinyl estradiol; HR, hazard ratio; ICD‐9‐CM, International Classification of Diseases, Ninth Revision, Clinical Modification; IUD, intrauterine device; MRI, magnetic resonance imaging; NCAA, National Collegiate Athletic Association; OC, oral contraceptive; OR, odds ratio; RR, relative risk.

Study populations ranged from collegiate athletes and recreational alpine skiers to women identified through large administrative, insurance, prescription and registry databases. Participant ages generally ranged from 13 to 49 years, and sample sizes varied substantially across studies.

Definitions of OC exposure differed among studies. Exposure was ascertained through prescription records, insurance claims, medication codes, medical records or self‐report. Some studies evaluated combined estrogen‐progestin OCs, whereas others assessed OC use without specifying formulation or used broader definitions of hormonal therapy. Exposure windows ranged from current use or use within the preceding 6 or 12 months to prescription histories extending up to 5 years before the index event.

Seven studies provided directly reported effect estimates or sufficient comparative data to calculate crude ORs and were included in the quantitative synthesis [1, 5, 6, 7, 11, 20, 22]. Three studies were included only in the qualitative synthesis because they did not provide an appropriate comparison group or sufficient data to estimate the association between OC use and ACL injury or reconstruction [3, 15, 24]. Outcomes included clinically or imaging‐confirmed ACL injury and ACL reconstruction identified through operative registries, procedural codes or medical records.

### Risk of bias

After reassessment of confounding, no study was judged to have a low overall risk of bias. Seven comparative studies were classified as having some concerns, and the three case‐only studies were judged to be at high risk of bias for the association between OC use and ACL outcomes. No study was rated as having a very high overall risk of bias.

Agel et al. [1], DeFroda et al. [5], Fry et al. [6], Gray et al. [7], Herzog et al. [11], Ruedl et al. [22] and Rahr‐Wagner et al. [20] were classified as having some concerns. Arendt et al. [3], Lefevre et al. [15] and Wang et al. [24] were judged to be at high risk of bias because they lacked an uninjured or non‐ACL comparison group and therefore could not address confounding of the association of interest; none of these three studies contributed to the quantitative synthesis.

At the domain level, confounding was rated as having some concerns in all seven comparative studies because none measured individual sporting exposure, training volume, prevention‐program participation, prior knee injury or contraceptive indication and three estimates were unadjusted; confounding was rated as high risk in the three case‐only studies. All studies were judged to be at low risk for measurement of exposure, selection of participants, postexposure interventions and selection of the reported result. Measurement of outcomes was rated as having some concerns in DeFroda et al., Fry et al., Gray et al., Ruedl et al., Rahr‐Wagner et al. and Wang et al. Lefevre et al. was the only study judged to have some concerns regarding missing data.

Detailed domain‐level judgements and overall ROBINS‐E assessments are presented in Tables S2 and S3. Injury‐only studies and Wang et al. contribute a qualitative context, not comparative evidence of incident injury risk.

### Certainty of evidence assessment

The GRADE assessment indicated very low certainty of evidence for the primary adjusted or matched analysis of ACL reconstruction or operatively treated injury. Because all contributing studies were observational, the certainty of evidence was initially rated as low and was further downgraded for serious inconsistency because of substantial statistical heterogeneity (*I* ^2^ = 84%; *τ* ^2^ = 0.01) and variability in study populations, exposure definitions and outcome ascertainment. Serious imprecision was also identified: the pooled 95% CI was 0.77–1.02 and the 95% prediction interval was 0.66–1.19. Risk of bias was also judged serious because all four contributing studies had some concerns, mainly due to residual confounding by sporting exposure; because certainty was already very low, this did not change the overall rating.

The certainty of evidence for the age‐stratified findings was also rated as very low. This judgement reflected the observational nature of the evidence, variation in the magnitude of the reported associations, the limited number of studies providing comparable age‐specific estimates and the concentration of the available evidence among women aged 15–19 years.

Evidence regarding OC formulation, dose pattern, duration and timing of use was rated as very low certainty. The observational evidence was further limited by substantial heterogeneity in the contraceptive formulations, exposure windows and duration categories evaluated, as well as by limited subgroup data and the absence of sufficiently comparable estimates for quantitative pooling.

The sensitivity analysis adding the crude Fry reconstruction estimate yielded an estimate similar to that of the primary analysis (OR, 0.85; 95% CI, 0.74–0.98). However, the certainty of evidence remained very low because the contributing studies were observational, all had some concerns for confounding and substantial heterogeneity persisted (*I* ^2^ = 90%; *τ* ^2^ = 0.01; 95% prediction interval, 0.61–1.19). The complete GRADE assessment is presented in Table S4.

### OC use and outcome‐specific associations

Seven studies contributed to quantitative analyses [1, 5, 6, 7, 11, 20, 22]. The primary analysis included four adjusted or matched estimates for reconstruction or operatively treated injury [5, 7, 11, 20]. The pooled association was not statistically significant (OR, 0.89; 95% CI, 0.77–1.02; *p* = 0.08), with substantial heterogeneity (*I* ^2^ = 84%; *τ* ^2^ = 0.01). The 95% prediction interval, 0.66–1.19, included associations on either side of the null (Figure 2a).

**Figure 2:**
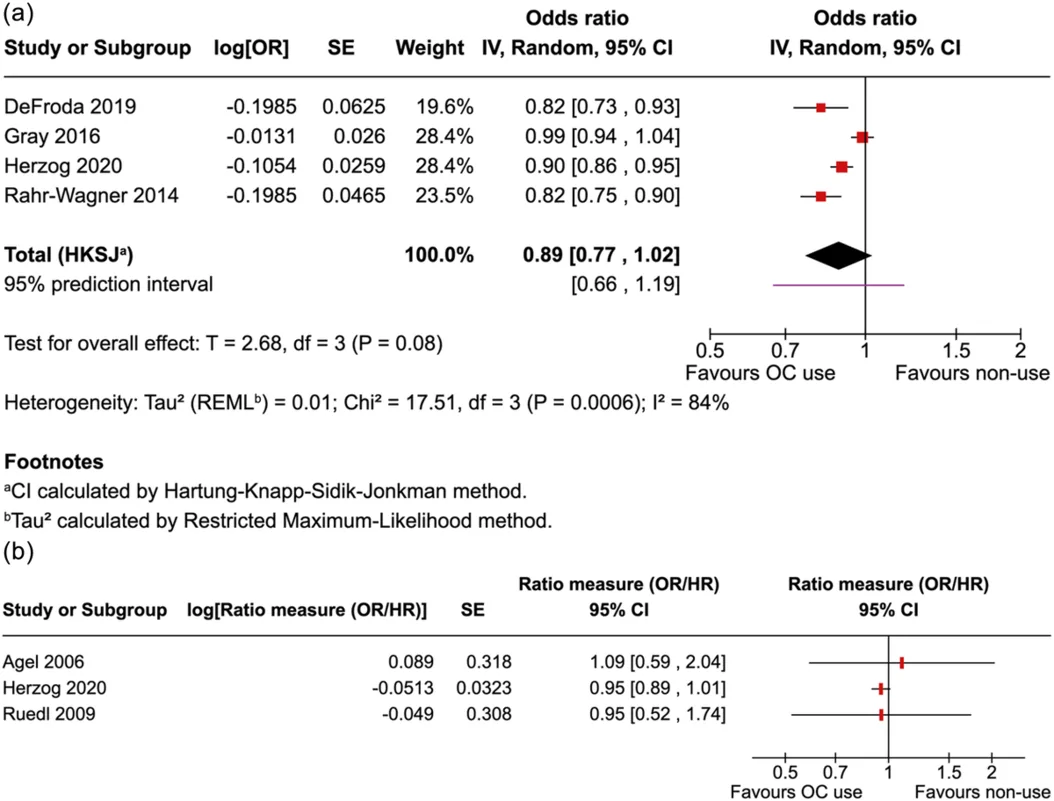
Association between oral contraceptive use and ACL injury or reconstruction. Figure 2. Outcome‐specific associations between OC use and ACL outcomes. Panel (a) presents the primary meta‐analysis of adjusted or matched estimates for ACL reconstruction or operatively treated ACL injury. Panel (b) presents the clinically identified ACL injury estimates and Herzog's active‐comparator new‐user cohort estimate separately, without pooling. The cohort outcome included diagnosis or reconstruction. RevMan displays symmetric log‐scale CIs reconstructed from rounded estimates and standard errors; these may differ from the published limits retained in Table 1. For DeFroda, the published CI is 0.72–0.92 and the plotted CI is 0.73–0.93; for Herzog's case‐control estimate, the published CI is 0.85–0.94 and the plotted CI is 0.86–0.95. ACL, anterior cruciate ligament; CI, confidence interval; HKSJ, Hartung‐Knapp‐Sidik‐Jonkman; IV, inverse variance; OC, oral contraceptive; OR, odds ratio; REML, restricted maximum likelihood; SE, standard error.

For clinically identified ACL injury, the crude estimates from Agel et al. (OR, 1.09; 95% CI, 0.59–2.04) and Ruedl et al. (OR, 0.95; 95% CI, 0.52–1.74) were not statistically significant [1, 22]. Herzog et al.'s active‐comparator new‐user cohort likewise found no clear association (adjusted HR, 0.95; 95% CI, 0.89–1.01) [11]. The cohort defined ACL injury through diagnostic codes or reconstruction, whereas its case‐control replication required reconstruction. These estimates are displayed separately without pooling (Figure 2b).

### Age‐stratified findings

Three studies reported findings for women aged 15–19 years [5, 6, 7]. Gray et al. found a statistically significant association between OC use and lower odds of ACL reconstruction in this age group (adjusted OR, 0.82; 95% CI, 0.75–0.91) [7]. DeFroda et al. likewise reported lower odds of ACL reconstruction among OC users aged 15 to 19 years (OR, 0.37; 95% CI, 0.27–0.50) [5].

Fry et al. reported ACL reconstruction incidences of 0.101% among OC users and 0.118% among nonusers aged 15–19 years [6]. However, the study did not report an age‐specific comparative effect estimate or the corresponding denominators. Because of differences in outcome ascertainment and the limited availability of comparable effect estimates, no pooled analysis was performed. The estimates reported by Gray et al. and DeFroda et al. are presented in Figure 3.

**Figure 3:**
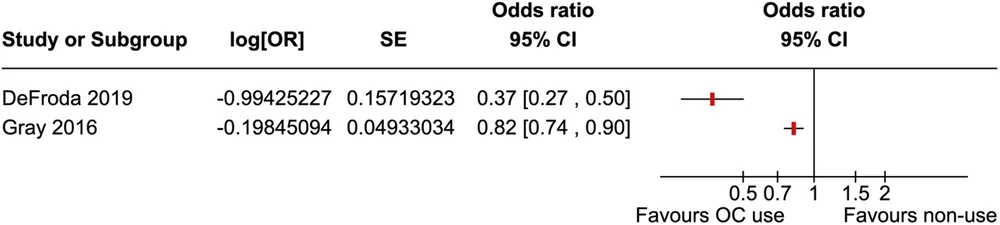
Age‐stratified association between oral contraceptive use and ACL reconstruction. Figure 3. Study‐specific effect estimates for the association between oral contraceptive use and anterior cruciate ligament (ACL) reconstruction among women aged 15–19 years. Gray et al. reported an adjusted odds ratio (OR) from a matched case‐control analysis, and DeFroda et al. reported an OR from a matched database analysis; both outcomes were ACL reconstruction. Squares represent the study‐specific effect estimates, and horizontal lines represent the corresponding 95% CIs. The vertical line at OR = 1 indicates no association. The published Gray CI is 0.75–0.91; the RevMan plot shows a symmetric log‐scale CI of 0.74–0.90 from the entered point estimate and standard error. No pooled estimate was calculated because of differences in outcome ascertainment, exposure definitions and analytical methods across studies. ACL, anterior cruciate ligament; CI, confidence interval; OC, oral contraceptive; OR, odds ratio.

### OC formulation and duration of use

Reporting of OC formulation, dose pattern, duration of use and timing of exposure varied across studies and was therefore summarised narratively (Table S6). Gray et al. [7] reported subgroup analyses according to formulation, dosing pattern and duration of use. In that study, neither progestin‐only OCs (OR, 0.89; 95% CI, 0.69–1.14) nor combined estrogen‐progestin formulations (OR, 0.99; 95% CI, 0.94–1.04) were significantly associated with ACL reconstruction. Monophasic formulations had an OR of 0.95 (95% CI, 0.90–1.01), whereas triphasic formulations had an OR of 1.06 (95% CI, 0.98–1.14). Use for 90 days or less was associated with lower odds of ACL reconstruction (OR, 0.89; 95% CI, 0.81–0.99), whereas use for more than 90 days was not (OR, 1.02; 95% CI, 0.96–1.07).

Rahr‐Wagner et al. evaluated timing and cumulative duration of OC use [20]. Compared with never users, lower risk estimates were reported for ever users (RR, 0.82; 95% CI, 0.75–0.90), recent users (RR, 0.81; 95% CI, 0.72–0.89) and long‐term users (RR, 0.80; 95% CI, 0.74–0.91), whereas the estimate for new users was RR 0.89 (95% CI, 0.76–1.05). According to cumulative duration, the reported estimates were RR 0.83 (95% CI, 0.74–0.94) for 1 year, RR 0.78 (95% CI, 0.68–0.90) for 2 years, RR 0.75 (95% CI, 0.64–0.90) for 3 years, RR 0.81 (95% CI, 0.69–0.96) for 4 years and RR 0.88 (95% CI, 0.77–1.01) for more than 4 years of use.

Herzog et al. evaluated low‐dose combined OCs, defined as estrogen‐progestin formulations containing 35 μg or less of ethinyl estradiol [11]. In the active‐comparator new‐user cohort analysis, the adjusted HR for ACL injury (diagnosis or reconstruction) was 0.95 (95% CI, 0.89–1.01). In the replicated case‐control analyses, the reported ORs were 0.96 (95% CI, 0.94–0.99) for the 1‐year look‐back window and 0.90 (95% CI, 0.85–0.94) for the 5‐year look‐back window.

Fry et al. reported ACL reconstruction incidence according to systemic hormonal contraceptive use and specific OC formulations [6]. Incidence was 0.079% (95% CI, 0.075%–0.083%) among systemic hormonal contraceptive users, 0.088% (95% CI, 0.081%–0.095%) among total OC users and 0.120% (95% CI, 0.118%–0.121%) among noncontraceptive users. By formulation, the reported incidences were 0.030% (95% CI, 0.020%–0.043%) for norethindrone‐only users, 0.093% (95% CI, 0.082%–0.105%) for norgestimate–ethinyl estradiol users, 0.099% (95% CI, 0.088%–0.112%) for norethindrone–ethinyl estradiol users and 0.096% (95% CI, 0.076%–0.119%) for drospirenone–ethinyl estradiol users.

Other included studies provided limited data on OC formulation or duration. DeFroda et al. defined exposure as combination OC use within 1 year before ACL injury but did not report formulation‐ or duration‐specific estimates [5]. Ruedl et al. assessed self‐reported OC use but did not record contraceptive type or duration [22]. Agel et al. evaluated hormonal therapy broadly and did not provide OC‐specific formulation or duration analyses [1]. Lefevre et al. described contraceptive types among ACL tear cases, but the study did not include a non‐ACL control group [15]. Wang et al. identified OC exposure using drug codes among patients undergoing ACL reconstruction but did not provide incident ACL injury risk estimates according to formulation or duration [24].

No meta‐analysis was performed for formulation, dose pattern, duration or timing of OC exposure because of differences in exposure definitions, comparator groups, outcome definitions and reporting formats across studies.

### Sensitivity analysis

Five studies were included in the sensitivity analysis adding Fry's crude reconstruction estimate to the four adjusted or matched estimates [5, 6, 7, 11, 20]. The pooled OR was 0.85 (95% CI, 0.74–0.98; *p* = 0.03), with substantial heterogeneity (*I* ^2^ = 90%; *τ* ^2^ = 0.01). The 95% prediction interval, 0.61–1.19, included the null (Figure 4). The original mixed‐outcome seven‐study synthesis yielded OR 0.86 (95% CI, 0.77–0.96) (*τ* ^2^ = 0.01; *I* ^2^ = 85%; 95% prediction interval, 0.65–1.14) and is retained only as a sensitivity analysis.

**Figure 4:**
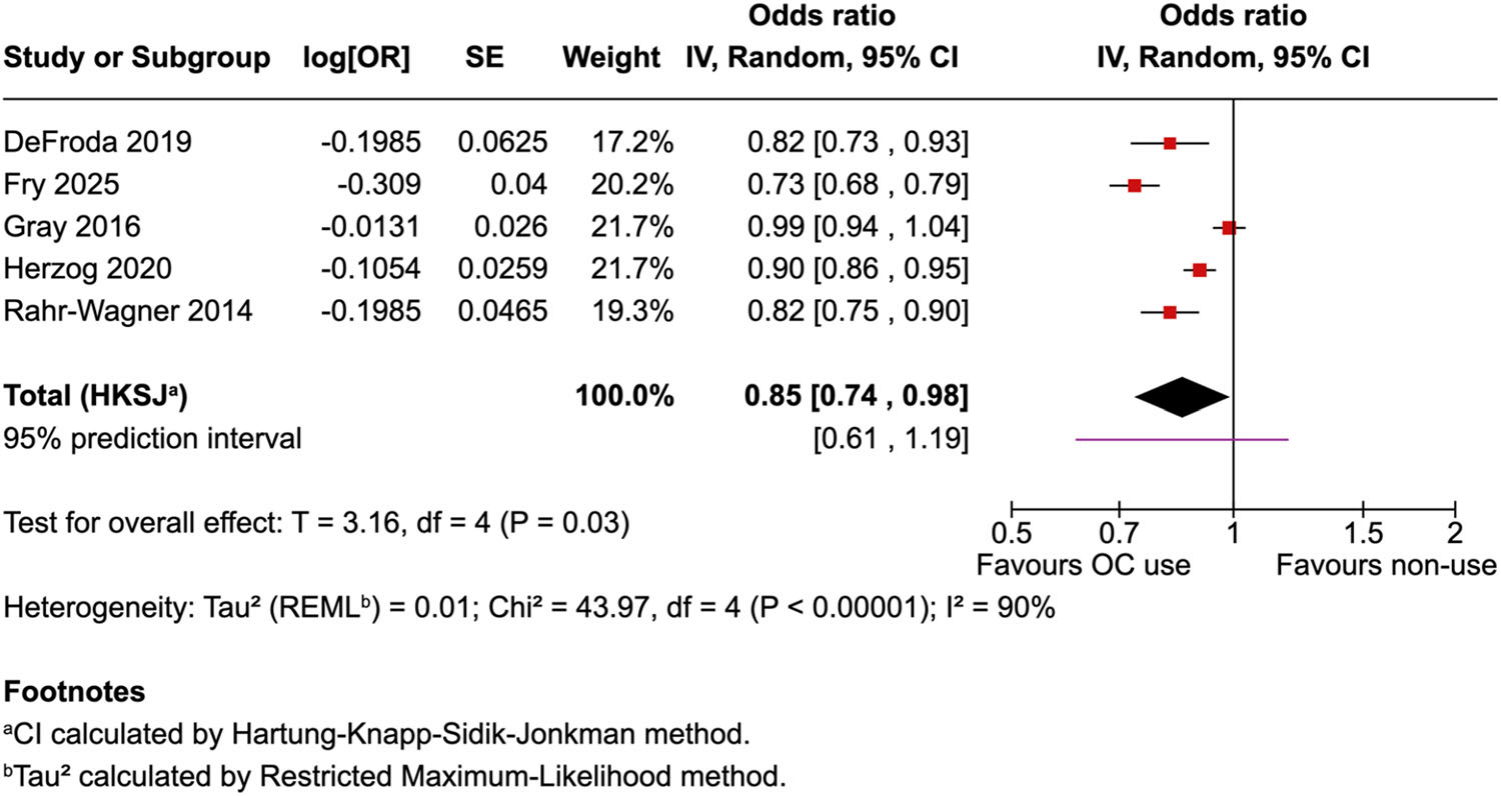
Sensitivity analysis including the crude fry reconstruction estimate. Figure 4. Forest plot of the sensitivity analysis adding the crude Fry estimate to the primary adjusted or matched reconstruction or operatively treated injury analysis. Study‐specific ORs were pooled using a random‐effects generic inverse variance model, REML estimation of between‐study variance, and HKSJ confidence intervals. The pooled OR was 0.85 (95% CI, 0.74–0.98; *p* = 0.03; *I* ^2^ = 90%; *τ* ^2^ = 0.01). The 95% prediction interval was 0.61–1.19. Reconstructed study‐level CIs differ slightly from published limits as explained in Figure 2. ACL, anterior cruciate ligament; CI, confidence interval; HKSJ, Hartung‐Knapp‐Sidik‐Jonkman; IV, inverse variance; OC, oral contraceptive; OR, odds ratio; REML, restricted maximum likelihood; SE, standard error.

In leave‐one‐out analyses of the primary model, the pooled CI excluded one only after omission of Gray. In the five‐study sensitivity model, three of five omissions produced CIs including one. These findings indicate study influence and limited robustness (Table S5).

## DISCUSSION

This systematic review and meta‐analysis found no statistically significant association in the primary adjusted or matched analysis of ACL reconstruction or operatively treated injury (OR, 0.89; 95% CI, 0.77–1.02). Substantial heterogeneity and very low certainty of evidence limit interpretation. Adding the crude Fry estimate produced a pooled CI excluding the null, but the prediction interval still included no association. Taken together, these findings support uncertainty about a possible association and do not establish that OC use causally prevents ACL injury.

The study by Herzog et al. is particularly informative because it evaluated the same research question using two epidemiologic designs [11]. A conventional case‐control analysis comparing ever users with never users reproduced a modest inverse association (OR, 0.90; 95% CI, 0.85–0.94). However, an active‐comparator, new‐user cohort analysis comparing OC initiators with intrauterine device users found no clear association (adjusted HR, 0.95; 95% CI, 0.89–1.01). The active‐comparator design better aligned the start of follow‐up and reduced differences related to contraceptive indication and health care‐seeking behaviour. This contrast suggests that part of the inverse association observed in conventional database studies may be attributable to selection, exposure misclassification or uncontrolled confounding.

The direction and magnitude of the pooled estimate are broadly consistent with previous reviews, although the interpretation of this evidence has evolved as larger database studies and more rigorous epidemiologic designs have become available. Herzberg et al. concluded that hormonal contraceptives might be associated with an approximately 20% reduction in ACL injury risk, while emphasising that the overall strength of evidence was low [10]. A subsequent review of soft‐tissue outcomes similarly found that the higher‐quality studies tended to favour OC use but characterised the strength of the evidence as weak [14]. In contrast, a broader systematic review published in 2023 concluded that evidence regarding combined hormonal contraceptives and nonbone musculoskeletal outcomes was sparse and insufficient to support their use for prevention of ACL tears or other musculoskeletal injuries [26].

More recently, Paska et al. reported a pooled risk ratio of 0.88 (95% CI, 0.77–1.01) across 8 studies, with very high heterogeneity (*I* ^2^ = 97.3%), and concluded that hormonal contraceptive use did not appear to reduce ACL injury risk [18]. The point estimate in that review is close to the primary pooled OR of 0.89 observed in the present analysis. Therefore, differences in significance across sensitivity analyses should not be interpreted as evidence that the two reviews reached biologically opposing conclusions. Such variation can reflect differences in eligibility criteria, effect measures, study weighting and statistical methods. The consistent signal across reviews is a small relative association whose causal meaning and clinical importance remain uncertain. The contribution is a refinement of existing evidence through an updated OC‐specific search, adjusted‐estimate prioritisation, outcome separation, prediction intervals, influence analyses and formal assessment of risk of bias and certainty. The evidence does not justify a change in clinical practice.

The contrast between athlete studies and administrative datasets limits applicability to female athletes. Collegiate‐athlete and recreational‐skier studies had null‐compatible estimates and fewer events, whereas several large databases reported lower odds of reconstruction [1, 5, 6, 7, 11, 20, 22]. Differences in statistical power, sporting exposure and treatment selection may explain this contrast.

Athletic participation is likely to be a particularly important source of residual confounding. OC users and nonusers may differ in sport, competitive level, training volume, prior injury, body mass index, socioeconomic factors, pregnancy history, health behaviours and access to preventive care. Most administrative datasets did not measure individual athlete‐exposure, participation in pivoting sports, use of neuromuscular prevention programs or mechanism of injury [5, 6, 7, 11, 20]. Adjustment for age and comorbidity cannot fully account for these differences. For this reason, confounding was rated as having some concerns in all comparative studies, and even adjusted estimates cannot exclude residual confounding [13].

ACL reconstruction is not equivalent to injury incidence: it also reflects treatment access, activity demands, surgeon recommendation and patient preference [5, 6, 7, 11, 20]. Heterogeneous exposure windows, prescriptions that do not establish adherence, and differing comparator groups further limit comparability. Separating outcomes and adjusted from crude estimates improves interpretation but does not eliminate residual confounding.

No consistent age, formulation or duration pattern identifies a subgroup likely to benefit [5, 6, 7, 11, 20]. Lower odds among adolescents in Gray and DeFroda contrast with higher odds in some older age groups in Gray, and Fry reported only unadjusted age‐specific incidences; nonrandomized formulation comparisons and the lack of a reproducible duration‐response pattern preclude recommending a specific regimen for ACL injury prevention.

A hormonal influence on ACL susceptibility remains biologically plausible. Estrogen receptors have been identified in ACL tissue, and experimental work suggests that estrogen may influence fibroblast activity and collagen metabolism [16]. Higher serum relaxin concentrations have been associated with ACL tears in collegiate athletes, and relaxin may promote collagen remodelling [4]. Clinical studies have also reported lower anterior tibial translation among OC users and variation in ACL injury occurrence across menstrual‐cycle phases [17, 27]. Suppression of ovulation and attenuation of endogenous hormonal fluctuations could therefore provide a potential mechanism for an effect of OCs. Consistent with this mechanistic hypothesis, a recent prospective cohort found lower estrogen, progesterone and relaxin levels and reduced peak knee adduction among hormonal contraceptive users, although overall musculoskeletal injury counts did not differ significantly between users and nonusers [23].

Nevertheless, biological plausibility is not evidence of clinical efficacy. Studies of menstrual‐cycle phase, hormone concentrations and knee laxity have produced inconsistent results, and laxity is an intermediate outcome that may not translate directly into injury reduction [10, 14]. Hormonal effects may also differ according to estrogen dose, progestin type, regimen, adherence, ovulatory suppression and individual endocrine response. The lack of a consistent association in the active‐comparator analysis and the inconsistent age, formulation and duration findings indicate that mechanistic hypotheses cannot overcome the limitations of the observational epidemiologic evidence.

The present findings do not support initiating or selecting an OC solely to prevent ACL injury. The pooled association was modest, absolute risk reduction could not be estimated consistently and certainty of evidence was very low. Contraceptive decisions should continue to be based on contraceptive needs, gynaecologic indications, patient preferences and an individualised assessment of benefits and potential harms. Athletes already using OCs should not be advised that they are protected from ACL injury, and nonusers should not be pressured to initiate hormonal contraception for an unproven musculoskeletal benefit. This caution is particularly important for adolescents and is consistent with recent systematic reviews [18, 26].

In clinical and sporting settings, established injury‐reduction strategies should remain the priority. Neuromuscular programs incorporating strength, plyometric, balance, landing and change‐of‐direction training reduce the incidence of ACL injury, including noncontact injury in female athletes [25]. OC use, if chosen for independent reproductive or medical reasons, should be considered a patient‐specific exposure rather than a substitute for evidence‐based training, workload management and sport‐specific prevention.

This review has several strengths. The search included multiple bibliographic databases without language or date restrictions, the protocol was prospectively registered and study selection and risk‐of‐bias assessment were performed independently. The analysis used random‐effects models with REML estimation and HKSJ CIs, which are appropriate when the number of studies is small and between‐study heterogeneity is expected. The review also prioritised adjusted or matched estimates, reported prediction intervals and influence analyses, examined the influence of outcome definition and evaluated certainty using GRADE.

The findings should be considered in light of important limitations. All included studies were observational, and adjusted or matched estimates may still be affected by residual confounding. Crude estimates were confined to separate displays or sensitivity analyses. Statistical heterogeneity was substantial, and the small number of studies limited investigation of its sources and precluded reliable assessment of publication bias. OC exposure was heterogeneous and frequently lacked information about formulation, dose, adherence, switching and proximity to injury. ACL reconstruction was often used as a proxy for ACL injury, potentially introducing treatment‐selection bias. Sport type, athlete‐exposure, mechanism of injury, prior injury and participation in prevention programs were incompletely measured. Four of the sevem meta‐analysed studies used US administrative data from overlapping calendar periods: Gray and Herzog used commercial insurance claims [7, 11], DeFroda used the PearlDiver database [5] and Fry used the Colorado Health Data Compass [6]. The same women may therefore contribute to more than one dataset, but the extent of overlap cannot be quantified from aggregate reports. Any duplicated participants could overstate precision and the amount of independent evidence, and leave‐one‐out analysis does not fully resolve this dependence. The available aggregate data were insufficient to calculate comparable absolute effects. Data extraction by a single investigator may have increased the risk of transcription or coding errors. In addition, one reported risk ratio was treated as an approximation of the OR under a rare‐outcome assumption, adding methodological heterogeneity. Finally, subgroup findings for age, formulation and duration were based on few studies and should be considered exploratory.

Future studies should prioritise prospective athlete cohorts and active‐comparator new‐user designs, measure sporting exposure and key confounders, distinguish injury from subsequent reconstruction and report standardised contraceptive exposure and absolute risks.

## CONCLUSION

The primary adjusted or matched analysis did not establish a clear association between OC use and anterior cruciate ligament reconstruction or operatively treated injury; substantial heterogeneity, residual confounding and very low certainty of evidence preclude causal interpretation. Current evidence does not support initiating or selecting OCs for anterior cruciate ligament injury prevention. Well‐designed prospective studies with standardised exposure and outcome definitions are needed.

## AUTHOR CONTRIBUTIONS

Eiki Nicholas Kobayashi conceived and designed the study, performed data extraction and analysis and drafted the manuscript. Natalia Zampa Silva and Laura Amaral Coelho de Azevedo contributed to the literature search, study selection and risk‐of‐bias assessment. Maria Eugênia Cammarota de Abreu, Juliano Mangini Dias Malpaga, Diego Escudeiro de Oliveira and Pedro Baches Jorge supervised the work and critically revised the manuscript. All authors read and approved the final manuscript.

## CONFLICT OF INTEREST STATEMENT

The authors declare no conflicts of interest.

## ETHICS STATEMENT

Ethical approval was not required because this study was a systematic review of published data. This systematic review used data from previously published studies.

## Supporting information

Supporting File 1.

Supporting File 2.

Supporting File 3.

Supporting File 4.

Supporting File 5.

Supporting File 6.

## Data Availability

All data generated or analysed during this study are included in this article and its supplementary information files.
