# Supplementary material for: Oral contraceptive use and anterior cruciate ligament injury or reconstruction in women: A systematic review and meta‐analysis of very low‐certainty evidence
Source: J Exp Orthop. 2026 Oct 6;13(4):e70940. doi: 10.1002/jeo2.70940 (PMC13641180; doi:10.1002/jeo2.70940)
Supplement: Supplementary file 1 [file JEO2-13-e70940-s001.docx]

**Supplementary Table S3. Overall Risk-of-Bias Judgments for the Included Studies Using ROBINS-E**

| **Study result** | **Domain 1** | **Domain 2** | **Domain 3** | **Domain 4** | **Domain 5** | **Domain 6** | **Domain 7** | **Overall risk of bias** | **Predicted direction of bias** | **Threatens conclusions?** | **Comments** |
| --- | --- | --- | --- | --- | --- | --- | --- | --- | --- | --- | --- |
| Agel 2006 [19] | Some concerns | Low risk | Low risk | Low risk | Low risk | Low risk | Low risk | Some concerns | Towards null | No | Prospective injury-surveillance cohort; crude estimate |
| Arendt 2002 [20] | High risk | Low risk | Low risk | Low risk | Low risk | Low risk | Low risk | High risk | Towards null | No | Descriptive injury-only case series without an uninjured comparator |
| DeFroda 2019 [11] | Some concerns | Low risk | Low risk | Low risk | Low risk | Some concerns | Low risk | Some concerns | Towards benefit of (higher) exposure | No | Retrospective matched database study |
| Fry 2025 [13] | Some concerns | Low risk | Low risk | Low risk | Low risk | Some concerns | Low risk | Some concerns | Towards benefit of (higher) exposure | No | Retrospective comparative database study; crude estimate |
| Gray 2016 [14] | Some concerns | Low risk | Low risk | Low risk | Low risk | Some concerns | Low risk | Some concerns | Towards benefit of (higher) exposure | No | Matched case-control study using insurance claims |
| Herzog 2020 [12] | Some concerns | Low risk | Low risk | Low risk | Low risk | Low risk | Low risk | Some concerns | Towards null | No | Retrospective active-comparator new-user cohort (claims) with case-control replication |
| Lefevre 2013 [21] | High risk | Low risk | Low risk | Low risk | Some concerns | Low risk | Low risk | High risk | Towards null | No | Prospective injury-only case series; OC use was not the primary exposure |
| Ruedl 2009 [9] | Some concerns | Low risk | Low risk | Low risk | Low risk | Some concerns | Low risk | Some concerns | Towards null | No | Case-control study; crude estimate |
| Rahr-Wagner 2014 [10] | Some concerns | Low risk | Low risk | Low risk | Low risk | Some concerns | Low risk | Some concerns | Towards benefit of (higher) exposure | No | Registry-based case-control study |
| Wang 2023 [22] | High risk | Low risk | Low risk | Low risk | Low risk | Some concerns | Low risk | High risk | Towards harm of (higher) exposure | No | Retrospective case-only analysis within an ACL reconstruction cohort |

Supplementary Table S3. Overall risk-of-bias judgments for the association between oral contraceptive use and anterior cruciate ligament injury or reconstruction, assessed using the ROBINS-E tool. The overall judgment was based on the domain-level assessments and was classified as low risk, some concerns, high risk, very high risk, or no information. The table also presents the outcome or effect estimate assessed, the predicted direction of bias, whether the identified bias was considered likely to threaten the study conclusions, and relevant methodological comments. The rationale for Domain 1 (confounding) judgments is given in Supplementary Table S2.

Abbreviations: ACL, anterior cruciate ligament; CI, confidence interval; HC, hormonal contraceptive; HR, hazard ratio; OC, oral contraceptive; OR, odds ratio; RR, relative risk; ROBINS-E, Risk Of Bias In Non-randomized Studies of Exposures.
