# Supplementary material for: Oral contraceptive use and anterior cruciate ligament injury or reconstruction in women: A systematic review and meta‐analysis of very low‐certainty evidence
Source: J Exp Orthop. 2026 Oct 6;13(4):e70940. doi: 10.1002/jeo2.70940 (PMC13641180; doi:10.1002/jeo2.70940)
Supplement: Supplementary file 2 [file JEO2-13-e70940-s002.docx]

**Supplementary Table S5. Leave-one-out influence analyses**

| Analysis | Omitted study | OR | 95% CI | τ² | I² | CI includes 1 |
| --- | --- | --- | --- | --- | --- | --- |
| Primary adjusted or matched | None | 0.89 | 0.77–1.02 | 0.0065 | 84% | Yes |
| Primary adjusted or matched | DeFroda 2019 | 0.90 | 0.72–1.13 | 0.0070 | 88% | Yes |
| Primary adjusted or matched | Gray 2016 | 0.86 | 0.74–0.99 | 0.0020 | 52% | No |
| Primary adjusted or matched | Herzog 2020 | 0.88 | 0.67–1.16 | 0.0107 | 85% | Yes |
| Primary adjusted or matched | Rahr-Wagner 2014 | 0.91 | 0.73–1.13 | 0.0059 | 85% | Yes |
| Sensitivity including Fry | None | 0.85 | 0.74–0.98 | 0.0118 | 90% | No |
| Sensitivity including Fry | DeFroda 2019 | 0.86 | 0.70–1.05 | 0.0150 | 93% | Yes |
| Sensitivity including Fry | Gray 2016 | 0.82 | 0.71–0.94 | 0.0065 | 80% | No |
| Sensitivity including Fry | Herzog 2020 | 0.84 | 0.69–1.02 | 0.0149 | 90% | Yes |
| Sensitivity including Fry | Rahr-Wagner 2014 | 0.86 | 0.70–1.05 | 0.0152 | 93% | Yes |
| Sensitivity including Fry | Fry 2025 | 0.89 | 0.77–1.02 | 0.0065 | 84% | Yes |

Random-effects models with restricted maximum-likelihood estimation and Hartung–Knapp–Sidik–Jonkman confidence intervals, independently calculated from the log estimates and standard errors entered in the supplied RevMan Web analyses. I² is calculated from τ² and the typical within-study variance, consistent with the RevMan estimator. Rounded values are shown; the unrounded values were used in calculations.
