# Supplementary material for: Oral contraceptive use and anterior cruciate ligament injury or reconstruction in women: A systematic review and meta‐analysis of very low‐certainty evidence
Source: J Exp Orthop. 2026 Oct 6;13(4):e70940. doi: 10.1002/jeo2.70940 (PMC13641180; doi:10.1002/jeo2.70940)
Supplement: Supplementary file 3 [file JEO2-13-e70940-s004.docx]

**Supplementary Table S4. GRADE Assessment of Evidence on Oral Contraceptive Use and ACL Injury or Reconstruction**

| **Outcome** | Primary adjusted or matched analysis of ACL reconstruction or operatively treated ACL injury | Age-stratified association between oral contraceptive use and ACL injury or reconstruction | Association according to oral contraceptive formulation, dose pattern, duration, or timing of use | Sensitivity analysis of ACL reconstruction or operatively treated ACL injury including the crude Fry estimate |
| --- | --- | --- | --- | --- |
| **Study Design** | Four observational comparative studies | Observational administrative-database studies | Observational studies with heterogeneous exposure definitions and reporting formats | Observational comparative studies |
| **Risk of Bias** | Serious (all 4 studies had some concerns, mainly residual confounding by sporting exposure) | Serious (contributing studies had some concerns for confounding) | Serious (comparative studies had some concerns for confounding; case-only studies were at high risk) | Serious (all 5 studies had some concerns, mainly residual confounding by sporting exposure) |
| **Inconsistency** | Serious (substantial heterogeneity; I² = 84%; τ² = 0.01) | Serious (effect magnitude varied substantially; no pooled estimate) | Serious (marked heterogeneity in formulations, dose patterns, duration categories, and exposure windows) | Serious (substantial heterogeneity; I² = 90%; τ² = 0.01) |
| **Indirectness** | Not serious for the defined outcome (ACL reconstruction or operatively treated ACL injury) | Serious (evidence was primarily limited to women aged 15–19 years) | Serious (several studies did not directly compare specific formulations or durations; some used broader hormonal exposure definitions) | Not serious (outcome directly reflected ACL reconstruction or operatively treated ACL injury) |
| **Imprecision** | Serious (pooled OR, 0.89; 95% CI, 0.77–1.02; 95% prediction interval, 0.66–1.19) | Serious (only 2 directly comparable estimates; no pooled estimate) | Serious (limited subgroup data; several estimates crossed the null or were reported only as incidences) | Serious (pooled OR, 0.85; 95% CI, 0.74–0.98). The 95% prediction interval, 0.61–1.19, additionally indicates uncertainty across settings. |
| **Publication Bias** | Not formally assessable (4 studies) | Not formally assessable (3 studies) | Not formally assessable (<10 studies) | Not formally assessable (5 studies) |
| **Overall Certainty** | **Very low** | **Very low** | **Very low** | **Very low** |

Supplementary Table S4. Certainty of evidence was assessed using the Grading of Recommendations Assessment, Development and Evaluation (GRADE) approach. The primary domain comprises adjusted or matched estimates of ACL reconstruction or operatively treated ACL injury. The evaluation considered risk of bias, inconsistency, indirectness, imprecision, and publication bias; prediction intervals were incorporated into judgments of imprecision. Risk-of-bias judgments reflect the revised ROBINS-E assessment of confounding (Supplementary Tables S2 and S3). Because all contributing studies were observational, initial certainty was low and was downgraded when serious concerns were identified. Final certainty was classified as high, moderate, low, or very low.

Abbreviations: ACL, anterior cruciate ligament; CI, confidence interval; GRADE, Grading of Recommendations Assessment, Development and Evaluation; OC, oral contraceptive; OR, odds ratio.
