# Supplementary material for: Oral contraceptive use and anterior cruciate ligament injury or reconstruction in women: A systematic review and meta‐analysis of very low‐certainty evidence
Source: J Exp Orthop. 2026 Oct 6;13(4):e70940. doi: 10.1002/jeo2.70940 (PMC13641180; doi:10.1002/jeo2.70940)
Supplement: Supplementary file 4 [file JEO2-13-e70940-s005.docx]

**Supplementary Table S1. Database search strategies used for the systematic review**

| **Database** | **Complete search strategy** | **Records retrieved** |
| --- | --- | --- |
| PubMed | ("anterior cruciate ligament"[MeSH Terms] OR ("anterior"[All Fields] AND "cruciate"[All Fields] AND "ligament"[All Fields]) OR "anterior cruciate ligament"[All Fields] OR ("anterior cruciate ligament"[MeSH Terms] OR ("anterior"[All Fields] AND "cruciate"[All Fields] AND "ligament"[All Fields]) OR "anterior cruciate ligament"[All Fields] OR "acl"[All Fields])) AND ("contraceptives oral"[Supplementary Concept] OR "contraceptives oral"[All Fields] OR "oral contraceptive"[All Fields] OR "contraceptives, oral"[MeSH Terms] OR ("contraceptives"[All Fields] AND "oral"[All Fields]) OR "oral contraceptives"[All Fields] OR ("oral"[All Fields] AND "contraceptive"[All Fields]) OR ("contraceptives oral"[Supplementary Concept] OR "contraceptives oral"[All Fields] OR "birth control pills"[All Fields] OR "contraceptives, oral"[MeSH Terms] OR ("contraceptives"[All Fields] AND "oral"[All Fields]) OR "oral contraceptives"[All Fields] OR ("birth"[All Fields] AND "control"[All Fields] AND "pills"[All Fields])) OR (("hormon"[All Fields] OR "hormonal"[All Fields] OR "hormonally"[All Fields] OR "hormonals"[All Fields] OR "hormone s"[All Fields] OR "hormones"[Pharmacological Action] OR "hormones"[Supplementary Concept] OR "hormones"[All Fields] OR "hormone"[All Fields] OR "hormones"[MeSH Terms] OR "hormons"[All Fields]) AND ("contracept"[All Fields] OR "contracepted"[All Fields] OR "contracepting"[All Fields] OR "contraception"[MeSH Terms] OR "contraception"[All Fields] OR "contraceptions"[All Fields] OR "contraceptive agents"[Pharmacological Action] OR "contraceptive agents"[Supplementary Concept] OR "contraceptive agents"[All Fields] OR "contraceptive"[All Fields] OR "contraceptive devices"[MeSH Terms] OR ("contraceptive"[All Fields] AND "devices"[All Fields]) OR "contraceptive devices"[All Fields] OR "contraceptive agents"[MeSH Terms] OR ("contraceptive"[All Fields] AND "agents"[All Fields]) OR "contraceptives"[All Fields] OR "contraceptive s"[All Fields] OR "contraceptively"[All Fields]))) | 80 |
| Scopus | TITLE-ABS-KEY ( ( Anterior Cruciate Ligament OR ACL ) AND ( Oral Contraceptive OR Birth Control Pills OR Hormonal Contraceptive ) ) | 10 |
| Embase | ('anterior cruciate ligament'/exp OR 'anterior cruciate ligament' OR (anterior AND cruciate AND ('ligament'/exp OR ligament)) OR 'acl'/exp OR acl) AND ('oral contraceptive'/exp OR 'oral contraceptive' OR (oral AND ('contraceptive'/exp OR contraceptive)) OR 'birth control pills' OR (('birth'/exp OR birth) AND ('control'/exp OR control) AND pills) OR 'hormonal contraceptive'/exp OR 'hormonal contraceptive' OR (hormonal AND ('contraceptive'/exp OR contraceptive))) | 159 |
| LILACS | (mh:("Ligamento Cruzado Anterior") OR tw:("ligamento cruzado anterior" OR "anterior cruciate ligament")) AND (mh:("Anticonceptivos Orales") OR tw:("anticonceptivos orales" OR "contraceptivos orais" OR "oral contraceptives")) AND instance:"lilacs" | 0 |

Supplementary Table S1. Database search strategies used for the systematic review. PubMed/MEDLINE, Scopus, and Embase were searched on 31 July 2026. LILACS was rerun on 16 September 2026 using an expanded DeCS-aligned strategy with Spanish, Portuguese, and English terms. The zero LILACS yield may reflect indexing or search-sensitivity limitations and should not be interpreted as evidence that no relevant Latin American literature exists. No restrictions regarding publication date or language were applied.

**Abbreviations:** ACL, anterior cruciate ligament; LILACS, Latin American and Caribbean Health Sciences Literature; MeSH, Medical Subject Headings.
