# Supplementary material for: Oral contraceptive use and anterior cruciate ligament injury or reconstruction in women: A systematic review and meta‐analysis of very low‐certainty evidence
Source: J Exp Orthop. 2026 Oct 6;13(4):e70940. doi: 10.1002/jeo2.70940 (PMC13641180; doi:10.1002/jeo2.70940)
Supplement: Supplementary file 5 [file JEO2-13-e70940-s006.docx]

**Supplementary Table S2. Domain-Level Risk-of-Bias Assessment of the Included Studies Using ROBINS-E**

| **Domain** | **Code** | **Agel 2006 [19]** | **Arendt 2002 [20]** | **DeFroda 2019 [11]** | **Fry 2025 [13]** | **Gray 2016 [14]** | **Herzog 2020 [12]** | **Lefevre 2013 [21]** | **Ruedl 2009 [9]** | **Rahr-Wagner 2014 [10]** | **Wang 2023 [22]** |
| --- | --- | --- | --- | --- | --- | --- | --- | --- | --- | --- | --- |
| *Domain 1 — Confounding* |  |  |  |  |  |  |  |  |  |  |  |
| Domain 1 — Confounding | 1.1 |  |  | WN | WN | WN |  |  | WN | WN | SN |
| Domain 1 — Confounding | 1.2 |  |  | Y | Y | Y |  |  | Y | Y | Y |
| Domain 1 — Confounding | 1.3 |  |  | N | N | N |  |  | N | N | N |
| Domain 1 — Confounding | 1.4 |  |  | N | N | N |  |  | N | N | N |
| **Domain 1 — Confounding** | **Judgement** |  |  | **Some concerns** | **Some concerns** | **Some concerns** |  |  | **Some concerns** | **Some concerns** | **High risk** |
| **Domain 1 — Confounding** | **Direction** |  |  | **Insufficient information** | **Insufficient information** | **Insufficient information** |  |  | **Insufficient information** | **Insufficient information** | **Insufficient information** |
| **Domain 1 — Confounding** | **Threat** |  |  | **No** | **No** | **No** |  |  | **No** | **No** | **No** |
| *Domain 1 — Confounding* |  |  |  |  |  |  |  |  |  |  |  |
| Domain 1 — Confounding | 1.1 | WN | SN |  |  |  | WN | SN |  |  |  |
| Domain 1 — Confounding | 1.2 | Y | Y |  |  |  | Y | Y |  |  |  |
| Domain 1 — Confounding | 1.3 | Y | Y |  |  |  | Y | Y |  |  |  |
| Domain 1 — Confounding | 1.4 | NA | NA |  |  |  | NA | NA |  |  |  |
| Domain 1 — Confounding | 1.5 | N | N |  |  |  | N | N |  |  |  |
| **Domain 1 — Confounding** | **Judgement** | **Some concerns** | **High risk** |  |  |  | **Some concerns** | **High risk** |  |  |  |
| **Domain 1 — Confounding** | **Direction** | **Insufficient information** | **Insufficient information** |  |  |  | **Insufficient information** | **Insufficient information** |  |  |  |
| **Domain 1 — Confounding** | **Threat** | **No** | **No** |  |  |  | **No** | **No** |  |  |  |
| *Domain 2 — Measurement of exposure* |  |  |  |  |  |  |  |  |  |  |  |
| Domain 2 — Measurement of exposure | 2.1 |  |  | Y | Y | Y |  |  | Y | Y | Y |
| Domain 2 — Measurement of exposure | 2.2 |  |  | N | N | N |  |  | N | N | N |
| Domain 2 — Measurement of exposure | 2.3 |  |  | NA | NA | NA |  |  | NA | NA | NA |
| Domain 2 — Measurement of exposure | 2.4 |  |  | NA | NA | NA |  |  | NA | NA | NA |
| **Domain 2 — Measurement of exposure** | **Judgement** |  |  | **Low risk** | **Low risk** | **Low risk** |  |  | **Low risk** | **Low risk** | **Low risk** |
| **Domain 2 — Measurement of exposure** | **Direction** |  |  | **Insufficient information** | **Insufficient information** | **Insufficient information** |  |  | **Insufficient information** | **Insufficient information** | **Insufficient information** |
| **Domain 2 — Measurement of exposure** | **Threat** |  |  | **No** | **No** | **No** |  |  | **No** | **No** | **No** |
| *Domain 2 — Measurement of exposure* |  |  |  |  |  |  |  |  |  |  |  |
| Domain 2 — Measurement of exposure | 2.1 | Y | Y |  |  |  | Y | Y |  |  |  |
| Domain 2 — Measurement of exposure | 2.2 | N | N |  |  |  | N | N |  |  |  |
| Domain 2 — Measurement of exposure | 2.3 | NA | NA |  |  |  | NA | NA |  |  |  |
| Domain 2 — Measurement of exposure | 2.4 | NA | NA |  |  |  | NA | NA |  |  |  |
| **Domain 2 — Measurement of exposure** | **Judgement** | **Low risk** | **Low risk** |  |  |  | **Low risk** | **Low risk** |  |  |  |
| **Domain 2 — Measurement of exposure** | **Direction** | **Insufficient information** | **Insufficient information** |  |  |  | **Insufficient information** | **Insufficient information** |  |  |  |
| **Domain 2 — Measurement of exposure** | **Threat** | **No** | **No** |  |  |  | **No** | **No** |  |  |  |
| *Domain 3 — Selection of participants* |  |  |  |  |  |  |  |  |  |  |  |
| Domain 3 — Selection of participants | 3.1 | Y | Y | Y | Y | Y | Y | Y | Y | Y | Y |
| Domain 3 — Selection of participants | 3.2 | NA | NA | NA | NA | NA | NA | NA | NA | NA | NA |
| Domain 3 — Selection of participants | 3.3 | N | N | N | N | N | N | N | N | N | N |
| Domain 3 — Selection of participants | 3.4 | NA | NA | NA | NA | NA | NA | NA | NA | NA | NA |
| Domain 3 — Selection of participants | 3.5 | NA | NA | NA | NA | NA | NA | NA | NA | NA | NA |
| Domain 3 — Selection of participants | 3.6 | NA | NA | NA | NA | NA | NA | NA | NA | NA | NA |
| Domain 3 — Selection of participants | 3.7 | NA | NA | NA | NA | NA | NA | NA | NA | NA | NA |
| **Domain 3 — Selection of participants** | **Judgement** | **Low risk** | **Low risk** | **Low risk** | **Low risk** | **Low risk** | **Low risk** | **Low risk** | **Low risk** | **Low risk** | **Low risk** |
| **Domain 3 — Selection of participants** | **Direction** | **Insufficient information** | **Insufficient information** | **Insufficient information** | **Insufficient information** | **Insufficient information** | **Insufficient information** | **Insufficient information** | **Insufficient information** | **Insufficient information** | **Insufficient information** |
| **Domain 3 — Selection of participants** | **Threat** | **No** | **No** | **No** | **No** | **No** | **No** | **No** | **No** | **No** | **No** |
| *Domain 4 — Post-exposure interventions* |  |  |  |  |  |  |  |  |  |  |  |
| Domain 4 — Post-exposure interventions | 4.1 | N | N | N | N | N | N | N | N | N | N |
| Domain 4 — Post-exposure interventions | 4.2 | NA | NA | NA | NA | NA | NA | NA | NA | NA | NA |
| **Domain 4 — Post-exposure interventions** | **Judgement** | **Low risk** | **Low risk** | **Low risk** | **Low risk** | **Low risk** | **Low risk** | **Low risk** | **Low risk** | **Low risk** | **Low risk** |
| **Domain 4 — Post-exposure interventions** | **Direction** | **Insufficient information** | **Insufficient information** | **Insufficient information** | **Insufficient information** | **Insufficient information** | **Insufficient information** | **Insufficient information** | **Insufficient information** | **Insufficient information** | **Insufficient information** |
| **Domain 4 — Post-exposure interventions** | **Threat** | **No** | **No** | **No** | **No** | **No** | **No** | **No** | **No** | **No** | **No** |
| *Domain 5 — Missing data* |  |  |  |  |  |  |  |  |  |  |  |
| Domain 5 — Missing data | 5.1 | Y | Y | Y | Y | Y | Y | PY | Y | Y | Y |
| Domain 5 — Missing data | 5.2 | Y | Y | Y | Y | Y | Y | PY | Y | Y | Y |
| Domain 5 — Missing data | 5.3 | Y | Y | Y | Y | Y | Y | PY | Y | Y | Y |
| Domain 5 — Missing data | 5.4 | NA | NA | NA | NA | NA | NA | PN | NA | NA | NA |
| Domain 5 — Missing data | 5.5 | NA | NA | NA | NA | NA | NA | PN | NA | NA | NA |
| Domain 5 — Missing data | 5.6 | NA | NA | NA | NA | NA | NA | WY | NA | NA | NA |
| Domain 5 — Missing data | 5.7 | NA | NA | NA | NA | NA | NA | N | NA | NA | NA |
| Domain 5 — Missing data | 5.8 | NA | NA | NA | NA | NA | NA | Y | NA | NA | NA |
| Domain 5 — Missing data | 5.9 | NA | NA | NA | NA | NA | NA | PY | NA | NA | NA |
| Domain 5 — Missing data | 5.10 | NA | NA | NA | NA | NA | NA | NA | NA | NA | NA |
| **Domain 5 — Missing data** | **Judgement** | **Low risk** | **Low risk** | **Low risk** | **Low risk** | **Low risk** | **Low risk** | **Some concerns** | **Low risk** | **Low risk** | **Low risk** |
| **Domain 5 — Missing data** | **Direction** | **Insufficient information** | **Insufficient information** | **Insufficient information** | **Insufficient information** | **Insufficient information** | **Insufficient information** | **Insufficient information** | **Insufficient information** | **Insufficient information** | **Insufficient information** |
| **Domain 5 — Missing data** | **Threat** | **No** | **No** | **No** | **No** | **No** | **No** | **No** | **No** | **No** | **No** |
| *Domain 6 — Measurement of outcome* |  |  |  |  |  |  |  |  |  |  |  |
| Domain 6 — Measurement of outcome | 6.1 | N | N | PY | PY | PY | N | N | PY | PY | PY |
| Domain 6 — Measurement of outcome | 6.2 | Y | Y | Y | Y | Y | Y | Y | Y | Y | Y |
| Domain 6 — Measurement of outcome | 6.3 | N | N | N | N | N | N | N | N | N | N |
| **Domain 6 — Measurement of outcome** | **Judgement** | **Low risk** | **Low risk** | **Some concerns** | **Some concerns** | **Some concerns** | **Low risk** | **Low risk** | **Some concerns** | **Some concerns** | **Some concerns** |
| **Domain 6 — Measurement of outcome** | **Direction** | **Insufficient information** | **Insufficient information** | **Insufficient information** | **Insufficient information** | **Insufficient information** | **Insufficient information** | **Insufficient information** | **Insufficient information** | **Insufficient information** | **Insufficient information** |
| **Domain 6 — Measurement of outcome** | **Threat** | **No** | **No** | **No** | **No** | **No** | **No** | **No** | **No** | **No** | **No** |
| *Domain 7 — Selection of reported result* |  |  |  |  |  |  |  |  |  |  |  |
| Domain 7 — Selection of reported result | 7.1 | Y | Y | Y | Y | Y | Y | Y | Y | Y | Y |
| Domain 7 — Selection of reported result | 7.2 | NA | NA | NA | NA | NA | NA | NA | NA | NA | NA |
| Domain 7 — Selection of reported result | 7.3 | N | N | N | N | N | N | N | N | N | N |
| Domain 7 — Selection of reported result | 7.4 | N | N | N | N | N | N | N | N | N | N |
| Domain 7 — Selection of reported result | 7.5 | N | N | N | N | N | N | N | N | N | N |
| **Domain 7 — Selection of reported result** | **Judgement** | **Low risk** | **Low risk** | **Low risk** | **Low risk** | **Low risk** | **Low risk** | **Low risk** | **Low risk** | **Low risk** | **Low risk** |
| **Domain 7 — Selection of reported result** | **Direction** | **Towards null** | **Towards null** | **Towards benefit** | **Towards benefit** | **Towards benefit** | **Towards null** | **Towards null** | **Towards null** | **Towards benefit** | **Towards harm** |
| **Domain 7 — Selection of reported result** | **Threat** | **No** | **No** | **No** | **No** | **No** | **No** | **No** | **No** | **No** | **No** |

Supplementary Table S2. Domain-level risk-of-bias judgments for the included studies according to the Risk Of Bias In Non-randomized Studies of Exposures (ROBINS-E) tool. The 7 domains assessed were bias due to confounding, bias in measurement of exposures, bias in selection of participants into the study or analysis, bias due to post-exposure interventions, bias due to missing data, bias in measurement of outcomes, and bias in selection of the reported result. Judgments were classified as low risk, some concerns, high risk, very high risk, or no information. For Domain 1 (confounding), comparative studies were rated as having some concerns because individual sporting exposure, training volume, prevention-program participation, prior knee injury, and contraceptive indication were not measured (and 3 estimates were unadjusted); case-only studies without an uninjured or non-ACL comparison group were rated as high risk because they could not address confounding of the association of interest.

Abbreviations: ACL, anterior cruciate ligament; NA, not applicable; NI, no information; N, no; PN, probably no; PY, probably yes; SN, strong no; WN, weak no; Y, yes.
